# Supplementary material for: Vowel onset measures and their reliability, sensitivity and specificity: A systematic literature review
Source: PLoS One. 2024 May 2;19(5):e0301786. doi: 10.1371/journal.pone.0301786 (PMC11065290; doi:10.1371/journal.pone.0301786)
Supplement: S1 File — (DOCX) [file pone.0301786.s002.docx]

**S1: Vowel Onset Systematic Review Protocol**

**Title**

**Identification:** Vowel onset measures and their reliability, sensitivity and specificity: A systematic literature review

**Registration** 
**Statement:** This protocol has been registered through the PROPERO International Prospective Register for Systematic Reviews (registration number CRD42021266384).

**Authors**

**Contacts:** 
Corresponding author: Antonia Chacon 
[antonia.chacon@sydney.edu.au](mailto:antonia.chacon@sydney.edu.au)

Author affiliations:

1. Voice Research Laboratory/ Doctor Liang Voice Program,  
   Faculty of Medicine and Health, Sydney School of Health Sciences 
   The University of Sydney

**Email**:  
Associate Professor Cate Madill (CM)- [cate.madill@sydney.edu.au](mailto:cate.madill@sydney.edu.au) 
Professor Michael Döllinger (MD)- [michael.doellinger@uk-erlangen.de](mailto:michael.doellinger@uk-erlangen.de)  
Dr Duy Duong Nguyen (DN)- [duong.nguyen@sydney.edu.au](mailto:duong.nguyen@sydney.edu.au) 
Mr Tomás Arias (TA)- [tomas.arias-vergara@uk-erlangen.de](mailto:tomas.arias-vergara@uk-erlangen.de)  
Mr John Holik (JH)- [john.holik@sydney.edu.au](mailto:john.holik@sydney.edu.au)  
Miss Antonia Chacon (AC)- [antonia.chacon@sydney.edu.au](mailto:antonia.chacon@sydney.edu.au) 
 
**Contribution plan:** AC is the guarantor. AC drafted the development of the systematic review search strategy with contributions from CM, MD and DN. All authors contributed to the development of the selection criteria. AC will conduct the initial literature search. Data extraction and data analysis will be divided amongst team members. DN is to offer statistical support. All authors will contribute to the writing of the review. All authors will read, provide feedback and approve the final manuscript.

**Support**

**Sources of support for review**: This review is partly funded through the Doctor Liang Voice Program, The University of Sydney through the funding of select research staff (AC, JH and DN). MD and TA are supported by the German Research Foundation (DFG).  
 
**Sponsor of review:** There is no sponsor for this systematic review. 

**Role of sponsor/ funder:** Funding of research staff.

**Introduction**

**Rationale:** It is estimated that 6.8% of the Australian population experience a voice disorder or some form of voicing difficulty during their adult life [1]. There are a range of symptoms which someone with a voice problem may encounter, including but not limited to a deterioration in voice quality, reduced voice functionality (i.e., decreased loudness, pitch control, vocal stamina) and increased sensations of vocal effort, discomfort and fatigue [2]. Further to this, a voice disorder will often have significant repercussions upon an individual’s physical, mental and emotional wellbeing; impacting work-related prospects and one’s ability to connect with others [3-5]. This burden extends to greater society, with voice disorders incurring major costs to healthcare and industry; particularly when these problems aren’t assessed, diagnosed and managed efficiently [6]. Novel voice assessment methods with great reliability, sensitivity and specificity are therefore needed for early detection and accurate classification of voice disorders, so that treatment can be administered in a timely manner to prevent more advanced diseases.

Traditionally, the assessment of the voice and evaluation of voice rehabilitative outcomes has focused upon voice quality [7, 8] and patient-reported outcomes [9] as measures of voice function and efficiency. Most current voice assessment methods focus on steady-state phonation while the perceptual and instrumental evaluation of the very first moments of voice production have not been investigated in depth. The initiation of voicing has been regarded as related to the quality of voice production [10]. It is suggested that examining voice onset can provide predictive information about the type of phonation which would be useful in the diagnosis of some types of voice disorders e.g., vocal hyperfunction [11].

Voice onset refers to the span of time between the release of a sound and the onset of voicing [12]. There are two types of voice onset, being that which occurs following the release of a consonant sound, and vowel onset without a preceding consonant. Vowel-initial voice onset has been selected as the focus for this review, as it is more clinically relevant (with vowel production being an established and standardised voice assessment task) yet has been researched to a lesser extent than consonant-initial voice onset. Voice onset predicts the voice function that follows and as such, has been increasingly suggested as an effective means of assessing one’s voice and response to treatment [12, 13]. However, the means through which vowel-initial voice onset has been measured across the existing evidence base, other than visual imaging of the vocal folds, is highly variable, with some researchers measuring vowel onset through airflow [14], others through electroglottography [15], acoustically [16] or through a spectrogram of the acoustic signal [17]. Nonetheless, no literature yet exists which has synthesised and consolidated which measures of vowel-initial voice onset are the most specific and sensitive, the contexts in which these measures may best be used nor established a common language amongst vowel onset types and the implications of these upon vocal function.

**Objective:** The aim of this systematic review is to evaluate the sensitivity, specificity, and reliability of existing measures of vowel onset and the contexts in which these can be used effectively. To this end, the proposed systematic review will answer the following: What are the current methods of assessing vowel onset and the evidence for their reliability, sensitivity and specificity?

**Methods**

This review protocol has been written in line with the Preferred Reporting Items for Systematic reviews and Meta-Analyses (PRISMA) 2020 statement [18].

**Eligibility criteria:** Studies will be selected according to the criteria stipulated below.

*Study designs*: Nil study design limits will be enforced. We will include randomised controlled trials (RCTs), including cluster RCTs, controlled (non-randomised) clinical trials (CCTs) or cluster trials, interrupted time series (ITS) studies, controlled before-after (CBA) studies, prospective and retrospective comparative cohort studies, cross-sectional and case-control studies, case series and case reports.

*Participants:* We will include studies examining the human population without any age limits.

*Setting*: There will be no exclusion for type of setting in which the study takes place. Research occurring in both laboratory and clinical settings will be considered.

*Language:* We will include articles reported in English only.

*Years considered:* Articles of relevance will be searched from 1900 to present day.

*Types of studies:* Both grey and published literature will be included in the review. Published articles will only be included if they have been peer reviewed. Studies will be excluded if they are not available in English. The grey literature to be considered will include pre-print articles and dissertations.

**Information sources:** Literature search strategies will be conducted through Medline (OVID interface), Embase (OVID interface), Scopus, Web of Science, CINAHL, PubMed Central and IEEE Xplore. The literature search will be limited to the English language. ProQuest will also be searched to capture unpublished dissertations. To ensure literature saturation, we will scan the reference lists of included studies or relevant reviews identified through the search to perform ‘snowball searching’ to capture additional articles of relevance.

**Search Strategy:** The following databases will be searched: Medline, Embase, Scopus, Web of Science, CINAHL, PubMed Central, IEEE Xplore and ProQuest. Studies will be included provided they outline at least one method of assessing voice onset. Studies will be limited to those available in English and published between the year 1900 and present day. The specific search strategy to be applied has been developed in collaboration with members of the research team. A draft search strategy to be used in Medline can be found in Appendix I. The Medline search strategy will be adapted to the syntax and subject headings of the other abovementioned electronic databases to be searched.

Two independent reviewers will screen the retrieved articles against the eligibility criteria using the Covidence platform. A narrative synthesis of the main findings will include means of measuring voice onset and the reliability, sensitivity and specificity of these measures.  The review will provide an insight into existing literature pertaining to measures of voice onset and how these measures are applied in research/ laboratory-based and clinical contexts.

An initial search through the aforementioned electronic databases will be completed in accordance with the search strategy stipulations provided in Appendix I.  The key terms have been determined through discussions between four of the authors (AC, CM, MD and DN). A search for grey literature and unpublished studies will also be completed at this stage. Following this, all titles will be entered into the Covidence platform to facilitate the subsequent stages of review. After duplicate removal, all authors will undergo a process of title screening to determine which articles meet the inclusion criteria. Included articles will then undergo a process of full-text screening.

Following full-text screening, the reference list of selected reports and articles included from the full-text screening process will be appraised and screened for additional papers.  The titles and abstracts of all identified studies potentially eligible for inclusion in the review will be screened and the full text versions of included articles will be obtained. Authors of primary studies or reviews may be contacted for further information should this be required. To this end, the search strategy will involve snowball searching once sources of relevance have been found.

**Study Records**

**Data Management:** Literature search results will be uploaded to the Covidence platform to manage data, facilitate collaboration and document the review process over the course of the study. Citation abstracts and full text articles will be uploaded to Covidence.

A cloud-based team sharing platform will be used to document daily each stage of the review process, challenges encountered, and decisions made by individuals (ahead of raising these with the review team) and through team discussion. Information of relevance documented here will be recorded in the final systematic review.

**Selection Process:** As the search is completed, all identified records will be collated and uploaded into Covidence with duplicates removed from the pool of results. Study selection will be undertaken in two stages. Firstly, titles and abstracts will be screened by at least two independent reviewers for assessment against the inclusion criteria for the review. Studies and reports that potentially meet the inclusion criteria will be retrieved in full and their citation details imported into the Covidence systematic review software. The full text of selected citations will be assessed in detail against the inclusion criteria by two or more independent reviewers. Titles and abstracts that don’t meet the study criteria will be removed, with reasons for exclusion of full text papers that do not meet the inclusion criteria being recorded and reported in the systematic review. We will seek additional information from study authors where necessary to resolve questions about eligibility and will record the reasons for excluding articles. Any disagreements that arise between the reviewers at each stage of the selection process will be resolved through discussion or through involvement of a third reviewer. Neither of the review authors will be blind to the journal titles nor to the study authors and their associated institutions. The results of the search will be reported in full in the final systematic review and presented in the format of the PRISMA 2020 flow diagram.

**Data Collection:** Data will be extracted from papers included in the systematic review by two or more independent reviewers. A data extraction table will be used (see Appendix II).  The data extracted will include specific details about the study population (authors, year of publication, participant details); measure of voice onset (acoustic/ visual/ airflow/ electroglottography, data used, level of evidence); context (research/ clinical context); methods (study design, test measures and reference standards used, results- reliability/ sensitivity/ specificity); and key findings relevant to the review question, amongst other domains. The draft data extraction tool will be trialed on a small number of studies to ensure all relevant information is extracted.  This will be modified and revised as necessary during the process of extracting data from each included paper. Modifications will be detailed in the systematic review. Any disagreements that arise between the reviewers will be resolved through discussion or by involving a third reviewer. Authors of papers will be contacted to request missing or additional data, where required, however a time limit of five working days will be imposed upon their responses.

**Data Items:** We will extract data pertaining to level of evidence, reliability, sensitivity and specificity of measures of voice onset. We will record provided information pertaining to the age and number of participants involved in each study, as well as the specific tasks assessed, and outcomes examined across each of these groups. Study design will be recorded, as well as perceived study limitations that are relevant for the purposes of our systematic review. Results relating to effect size and significance of findings will be documented. Where any data deemed important is not reported in the study, the project authors will be contacted for further information.

**Outcomes and Prioritisation:** The primary outcome to be examined is the level of evidence for each type of voice onset measure identified. A secondary outcome includes examining types and evidence of different voice onsets and the application of these measures.

**Risk of bias in individual studies:** Assessing risk of bias in individual studies will be completed using the Cochrane Risk of Bias Tool (Table 8.5.a in the Cochrane Handbook for Systematic Reviews of Interventions). This tool will be used to assess the collected studies following the process of data extraction into the Covidence platform. The tool will provide a systematic means of assessing bias across the areas of randomisation, deviation from intended intervention (effects of assignment and adhering), incomplete outcome data, outcome measurement and result reporting. We will structure our assessment by describing procedures undertaken for each study across each tool domain. Each study will then be classed as high or low risk based upon its outcomes for risk of bias across each of the six domains. In cases where insufficient information can be gathered from the study pertaining to risk of bias, the study authors will be contacted for further information to clarify this.

**Data** 

**Synthesis:**  
*Criteria for quantitative synthesis:* It is assumed that the collected studies will demonstrate high levels of heterogeneity owing to the different types of signals (acoustic, spectrographic, electroglottographic and airflow-based) each will elicit, analysis algorithms used and general inter-study variation, rendering meta-analysis impossible. However, if selected studies are sufficiently homogeneous in terms of design, we will conduct meta-analyses using parametric or non-parametric tests depending on the distribution of data. Mixed-effects models will be used for longitudinal data. If correlation data are available, meta-analyses for correlation coefficients will be calculated using weighted mean correlation coefficients for the population effect size.

*If quantitative synthesis is not appropriate, describe the type of summary planned:* If similar studies are found, we will work closely with our team’s statistical expert (DN) to determine the most appropriate means of analysing the collected outcomes based upon our findings.

Our summary will be an integrative analysis involving both narrative and statistical techniques to reflect the anticipated diversity of evidence to be collected from this review. This will involve use of a summary table similar to that detailed in Appendix II to report our findings (please note- this table is likely to evolve and involve further data points as the review unfolds), followed by categorisation of data collected in similar concept areas and thematic analysis of findings. We will involve the statistical expert within our team to complete a statistical analysis of the collective study outcomes when required.

**Meta-biases:** We will determine whether any biases exist in the areas of publication/dissemination and outcome reporting to address the possibility of meta-biases. Potential publication/ dissemination bias will be determined through searching for similar results across large and small study effect estimates, and comparing results found between the published literature identified through electronic database searches, as compared to the grey, unpublished literature found through ProQuest. Selective outcome reporting bias will be determined by comparing outcomes between the study protocol (where available) and published report; or, where the protocol isn’t accessible, comparing the published report with the article’s methods and results sections. This will also be supplemented by use of the Outcome Reporting Bias in Trials (ORBIT) tool where applicable to assess the relevant trial studies collected for review.

**Confidence in cumulative evidence:** The strength of the body of evidence collected through our review will be assessed through the Grading of Recommendations Assessment, Development and Evaluation (GRADE) working group methodology. This will involve examining the quality of evidence through the domains of risk of bias, consistency, precision, directness and publication bias. Following this evaluation, the team will determine whether the quality of the research may be deemed as high (i.e. very unlikely that further research will change our confidence in the estimate of effect), moderate (i.e. likely that further research will have an impact on our confidence in the estimate of effect and may change the estimate), low (i.e. very likely that further research will have an important impact on our confidence in the estimate of effect and is likely to change the estimate), or very low (i.e. very uncertain about the estimate of effect).

**Conflicts of interest**

The authors declare no conflict of interests.

**References**

1. Russell, A., J. Oates, and K. Greenwood, *Prevalence of self-reported voice problems in the general population in South Australia.* Advances in Speech Language Pathology, 2005. **7**(1): p. 24-30.

2. Merrill, R.M., N. Roy, and J. Lowe, *Voice-related symptoms and their effects on quality of life.* Annals of Otology, Rhinology & Laryngology, 2013. **122**(6): p. 404-411.

3. Etter, N.M., J.C. Stemple, and D.M. Howell, *Defining the lived experience of older adults with voice disorders.* Journal of Voice, 2013. **27**(1): p. 61-67.

4. Cohen, S.M., et al., *The impact of laryngeal disorders on work‐related dysfunction.* The Laryngoscope, 2012. **122**(7): p. 1589-1594.

5. Andrea, M., M. Andrea, and M.L. Figueira, *Self-perception of quality of life in patients with functional voice disorders: the effects of psychological and vocal acoustic variables.* European Archives of Oto-Rhino-Laryngology, 2018. **275**(11): p. 2745-2754.

6. Cohen, S.M., et al., *Delayed otolaryngology referral for voice disorders increases health care costs.* The American journal of medicine, 2015. **128**(4): p. 426. e11-426. e18.

7. Faham, M., et al., *Acoustic voice quality index as a potential tool for voice screening.* Journal of Voice, 2019.

8. Gillespie, A.I., et al., *An examination of pre-and posttreatment acoustic versus auditory perceptual analyses of voice across four common voice disorders.* Journal of Voice, 2018. **32**(2): p. 169-176.

9. Pestana, P.M., S. Vaz-Freitas, and M.C. Manso, *Prevalence of voice disorders in singers: systematic review and meta-analysis.* Journal of voice, 2017. **31**(6): p. 722-727.

10. Miller, R., *The mechanisms of singing: coordinating physiology and acoustics of singing*, in *Vocal arts medicine: The care and prevention of professional voice disorders*, M.S. Benninger, B.H. Jacobson, and A.F. Johnson., Editors. 1994, Thieme: New York, NY. p. 61–71.

11. Cooke, A., et al., *Characteristics of vocal fold adduction related to voice onset.* J Voice, 1997. **11**(1): p. 12-22.

12. McKenna, V.S., et al., *Voice onset time in individuals with hyperfunctional voice disorders: Evidence for disordered vocal motor control.* Journal of Speech, Language, and Hearing Research, 2020. **63**(2): p. 405-420.

13. Stepp, C.E., D.E. Sawin, and T.L. Eadie, *The relationship between perception of vocal effort and relative fundamental frequency during voicing offset and onset.* 2012.

14. DeJonckere, P.H. and J. Lebacq, *In vivo quantification of the intraglottal pressure: modal phonation and voice onset.* Journal of Voice, 2020. **34**(4): p. 645. e19-645. e39.

15. Rae, R.C., *Measures of voice onset time: A methodological study*. 2018, Bowling Green State University.

16. Abramson, A.S. and D.H. Whalen, *Voice Onset Time (VOT) at 50: Theoretical and practical issues in measuring voicing distinctions.* Journal of phonetics, 2017. **63**: p. 75-86.

17. Neiman, G.S., R.J. Klich, and E.M. Shuey, *Voice onset time in young and 70-year-old women.* Journal of Speech, Language, and Hearing Research, 1983. **26**(1): p. 118-123.

18. Page, M.J., et al., *Updating guidance for reporting systematic reviews: development of the PRISMA 2020 statement.* Journal of Clinical Epidemiology, 2021. **134**: p. 103-112.

**Appendix I: Search strategy**

MEDLINE (Ovid SP) Search Plan

The following search strategy will be adapted for each electronic database, with the limits of English language and publications from 1900 to present day applied. This search strategy is expected to evolve according to the results found based on the concept searches below:

To search ‘*Voice Onset’* terms ‘AND’ results of ‘*Evidence*’ terms ‘AND’ results of ‘*Measure*’ terms

| *Search Concept Area* | *Related search terms* |
| --- | --- |
| **Voice onset** | - Voice onset* - Vocal onset* - Voice onset tim* - Vocal onset tim* - Voice timing - Onset tim* - “VOT” - Breath* - Glottal attack* - Glottal stroke* - Simultan* - Glottal onset* - Breathy onset* - Simultan* onset* - Voice onset type* - Creaky onset* - Vowel onset* - Speech onset* - Speech tim* - Phonatory initiat* - Voicing initiat* - Tone onset* - Vocaliz* onset* - Vocal attack tim* - “VAT” - Phonation onset* - Voice Initiat* - Voice initation period* - “VIP” - Oscillat* onset* - Acoustic voice onset* - Oscillation initiat* - Vibrat* onset* - Intervocalic onset* |
| **Evidence** | - Eviden* - Data - Support* - Confirm* - Proof* - Prov* - Accura* - Level* of eviden* - Reliab* - Valid* - Sensitiv* - Specific* - Statistic* - Comparison* - Model* - Correlat* - Relation* - Predict* - Populat* - Appl* - Use* |
| **Measure** | - Measure* - Parameter* - Analys* - Analyz* - Assess* - Calculat* - Comput* - Quantif* - Evaluat* - Determin* - Identif* - Signal~~*~~ - Process* - Extract* - Waveform* - Spect* - Ampli* - Frequenc* - Intensit* - Duration* - Length* - Window* - Fram* - Laryngoscop* - Stroboscop* - High-speed* - Laryn* visuali* - Electroglotto* - EGG* - Airflow* - Air pressur* - Perceptual* - Laryn* resist* - Visual* - Auditor* - Acoustic* |

**Appendix II: Data extraction instrument**

| *Study title, authors, year* | *Study design and duration* | *Background* | *Study aims and objectives* | *Measurement of voice onset* | *Voice onset type/s explored* | *Setting* | *Participant details (age, number, setting, etc.)* | *Randomisation method (RCTs)* | *Factors used to minimise bias (if non-randomised)* | *Summary of method* | *Primary outcome measures (define and units of measurement)* | *Secondary outcome measures (define and units of measurement)* | *Statistical methods used* | *Key results/ data* | *Comments on research quality* | *Study limitations and confounders* | *Implications of study findings for purposes of review* | *Other useful studies referenced in article* |
| --- | --- | --- | --- | --- | --- | --- | --- | --- | --- | --- | --- | --- | --- | --- | --- | --- | --- | --- |
|  |  |  |  |  |  |  |  |  |  |  |  |  |  |  |  |  |  |  |
|  |  |  |  |  |  |  |  |  |  |  |  |  |  |  |  |  |  |  |
|  |  |  |  |  |  |  |  |  |  |  |  |  |  |  |  |  |  |  |
|  |  |  |  |  |  |  |  |  |  |  |  |  |  |  |  |  |  |  |
|  |  |  |  |  |  |  |  |  |  |  |  |  |  |  |  |  |  |  |
|  |  |  |  |  |  |  |  |  |  |  |  |  |  |  |  |  |  |  |
