# Supplementary material for: Vowel onset measures and their reliability, sensitivity and specificity: A systematic literature review
Source: PLoS One. 2024 May 2;19(5):e0301786. doi: 10.1371/journal.pone.0301786 (PMC11065290; doi:10.1371/journal.pone.0301786)
Supplement: S2 File — (DOCX) [file pone.0301786.s003.docx]

**S3: Database Search Strategies**

1. **Strategy for CINAHL, IEEE Xplore, ProQuest, PubMED, Scopus, Web of Science**

| **Search concept** | **Terms associated with each concept** |
| --- | --- |
| **Voice onset** | ‘Voice onset*' OR 'Vocal onset*' OR 'Voice tim*' OR 'Onset tim*' OR 'Glottal attack*' OR 'Glottal stroke*' OR 'Glottal onset*' OR 'Breath* onset*' OR 'Simultan* onset*' OR ‘Creaky onset*’ OR ‘Voice onset type*’ OR 'Vowel onset*' OR 'Speech onset*' OR ‘Speech tim*’ OR 'Phonat* initiat*' OR 'Voic* initiat*' OR 'Tone onset*' OR 'Vocali* onset*' OR 'Vocal attack tim*' OR 'Phonation onset*' OR 'Voice Initiat*' OR 'Oscillat* onset*' OR 'Acoustic voice onset*' OR 'Oscillation initiat*' OR 'Vibrat* onset' |
| **Evidence** | 'Eviden*' OR 'Data' OR ‘Eviden* level*’ OR ‘Accura*’ OR 'Reliab*' OR 'Valid*' OR 'Sensitiv*' OR 'Specific*' OR 'Correlat*' OR 'Predict' |
| **Measure** | ‘Measure*' OR 'Analy*' OR 'Assess*' OR ‘Calculat*’ OR 'Signal*' OR ‘Process*’ OR ‘Extract*’ OR 'Waveform*' OR 'Frequenc*' OR 'Laryngoscop*' OR 'Stroboscop*' OR 'High speed*' OR 'Laryn* visuali*' OR 'Electroglotto*' OR 'Perceptual*' OR 'Laryn* resist*' OR 'Acoustic*' OR ‘Airflow*’ OR ‘Air pressur*’ |

**All above concepts combined with ‘AND’**

**Limits placed on search:**
Language: English only
Population: Humans only (no animal studies)
Year of publication: 1900 onwards

1. **Strategy for OVID (EMBASE and MEDLINE)**
2. **Individual searching of ‘Voice Onset’ terms:**

- ‘Voice onset*'
- 'Vocal onset*'
- 'Voice tim*'
- 'Onset tim*'
- 'Glottal attack*'
- 'Glottal stroke*'
- 'Glottal onset*'
- 'Breath* onset*'
- 'Simultan* onset*'
- ‘Creaky onset*’
- ‘Voice onset type*’
- 'Vowel onset*'
- 'Speech onset*'
- ‘Speech tim*’
- 'Phonat* initiat*'
- 'Voic* initiat*'
- 'Tone onset*'
- 'Vocali* onset*'
- 'Vocal attack tim*'
- 'Phonation onset*'
- 'Voice Initiat*'
- 'Oscillat* onset*'
- 'Acoustic voice onset*'
- 'Oscillation initiat*'
- 'Vibrat* onset'

1. **Above ‘Voice Onset’ terms combined with ‘OR’**
2. **Individual searching of ‘Evidence’ terms:**

- 'Eviden*'
- 'Data'
- ‘Eviden* level*’
- ‘Accura*’
- 'Reliab*'
- 'Valid*'
- 'Sensitiv*'
- 'Specific*'
- 'Correlat*'
- 'Predict'

1. **Above ‘Evidence’ terms combined with ‘OR’**
2. **Individual searching of ‘Measure’ terms:**

- ‘Measure*'
- 'Analy*'
- 'Assess*'
- ‘Calculat*’
- 'Signal*'
- ‘Process*’
- ‘Extract*’
- 'Waveform*'
- 'Frequenc*'
- 'Laryngoscop*'
- 'Stroboscop*'
- 'High speed*'
- 'Laryn* visuali*'
- 'Electroglotto*'
- 'Perceptual*'
- 'Laryn* resist*'
- 'Acoustic*'
- ‘Airflow*’
- ‘Air pressur*’

1. **Above ‘Measure’ terms combined with ‘OR’**
2. **Term groupings (steps 2, 4 and 6) combined with ‘AND’**

**Limits placed on search:**
Language: English only
Population: Humans only (no animal studies)
Year of publication: 1900 onwards
