# Supplementary material for: Vowel onset measures and their reliability, sensitivity and specificity: A systematic literature review
Source: PLoS One. 2024 May 2;19(5):e0301786. doi: 10.1371/journal.pone.0301786 (PMC11065290; doi:10.1371/journal.pone.0301786)
Supplement: S3 File — (DOCX) [file pone.0301786.s004.docx]

**S4: Data Extraction Summary Table**

| **Study details** | **Voice onset measurement category/ies** | **Definition of voice onset** | **Study design** | **Sample size** | **Outcome measures** | **Summary of findings** |
| --- | --- | --- | --- | --- | --- | --- |
| Baken & Watson (2019): Research Note: Vocal Attack Time- Extended Analysis | Acoustic, physiological | Definition provided for Vocal Attack Time (VAT), not voice onset in general (see Table 2 for VAT definition) | Review paper with case examples | Not specified | Statistical analysis of differences in VAT values according to age and gender | For both American English and Cantonese speakers, men have longer VAT values than women. Statistical differences were found regarding gender and age and among Cantonese tone categories |
| Braunschweig, Flaschka, Schelhorn-Neise & Döllinger (2008): High-speed video analysis of the phonation onset, with an application to the diagnosis of functional dysphonias | Visual imaging, acoustic | Not provided | Solution validation with cross-sectional data | 71 | Parameter 'a', threshold value a_th_, and maximum sound pressure level L_max_ | Parameters a_th_ (myoelastic tonus of vocal folds) and L_max_ (maximum sound pressure level) were found to identify significant differences between pathological and normophonic voices |
| Choi, Oh & Choi (2015): Pattern Analysis of Voice Onset and Offset in Normal Adults Using High-Speed Digital Imaging: The Role of Arytenoid Cartilage Movements | Visual imaging | The interval from the initial movement of the vocal folds from resting position to the most open state of the glottis following the end of inspiration into regular vibration | Cross-sectional | 40 | Pattern analysis of voice onset and offset types and associated inter- and intra-rater reliability | Most adults demonstrated a type I onset pattern, with 30% demonstrating type II and a single participant with type III. High inter-rater and very high intra-rater reliability found. Normophonic males demonstrate immediate initiation of VF vibration after arytenoid adduction whereas normal females demonstrated a time delay. |
| Cohen, Cohen, Benyamini, Adi & Keshet (2019): Predicting glottal closure insufficiency using fundamental frequency contour analysis | Acoustic | Not provided | Cross-sectional | 38 | Settling time, slope and area under the fundamental frequency (F0) curve from phonation onset to settling time before and after injection medialization laryngoplasty | Settling time, slope and area under the curve are correlated with traditional acoustic voice assessments and clinical findings pre- and post- injection medialization laryngoplasty Two new parameters were defined to measure onset (Ss and As) which differentiate normophonic speakers from those with glottal closure insufficiency. |
| Cooke, Ludlow, Hallett & Selbie (1997): Characteristics of vocal fold adduction related to voice onset | Auditory perceptual, visual imaging | An indicator of vocal performance, characterised by perceptual categories of breathy, normal and hard. The onset of vocal fold adduction commences at the most open state of the glottis and is completed when the vocal process or folds first make contact and/or begin to vibrate | Cross-sectional | 10 | Normalised distance between vocal processes (anterior-posterior and left-right distances) and the time for their adduction | Speed and timing of vocal fold adduction differed among voice onset types with few gender differences. Significant linear relationships identified among onset types on measures of gesture duration, maximum velocity, time between completion of adduction and estimates of vocal fold stiffness |
| Freeman, Woo, Saxman & Murry (2012): A Comparison of Sung and Spoken Phonation Onset Gestures Using High-Speed Digital Imaging | Acoustic analysis, auditory perceptual, visual imaging | Not provided | Cross-sectional | 2 | Visual observations of high-speed image data; kymogram characteristics, mean number of small amplitude pre-phonatory oscillations (PPOs), timing data of onset gesture events | All gestures begin with small-amplitude oscillations at the medial edge of the vocal folds followed by vocal fold contact at the mid-point which marks the beginning of steady-state oscillation (SSO). Glottal area configuration varies amongst gestures and between subjects. Comparison of onset characteristics according to gender outlined. Three phonation onset events defined: Event A = time between maximum abduction and maximum adduction positioning; Event B = time between maximum adduction and beginning of PPO; Event C = Time between beginning of PPO and SSO |
| Ikuma, Kunduk, Fink & McWhorter (2016): A Spatiotemporal Approach to the Objective Analysis of Initiation and Termination of Vocal-fold Oscillation With High-speed Videoendoscopy | Visual imaging | Voice onset is defined according to the analysis approach taken in the study. Vocal initiation phase events observed through high speed videoendoscopy:  (A1) Adduction onset: vocal folds start to adduct  (A2): Adduction offset: vocal folds fully adducted to form pre-phonatory glottal closure  (A3): Oscillation onset: start of first oscillation  (A4): Oscillation reaching full length of vocal folds  (A5): Oscillation reaching steady-state amplitude (full width oscillation) | Solution validation with case examples | 3 | Vocal fold oscillation length and four transient events: oscillation onset, oscillation offset, attainment of full-length oscillation and loss of full-length oscillation | A detection algorithm can be developed based on high-speed imaging data to detect different transient phases related to phonation onset and offset. Glottal area, width, and length waveforms were described for example cases |
| Koike (1967): Experimental studies on vocal attack | Acoustic, aerodynamic, auditory perceptual, electromyography, visual imaging | Designates the onset of phonation with three classifications: the soft/ simultaneous, the breathy/ aspirate and the hard/ glottal onset | Cross-sectional | Different numbers per experiment; Acoustic: 33 Aerodynamic: 33 Electromyographic: 2 Cineradiographic: 12 | Acoustic: rise time of acoustic waves; Aerodynamic: air flow rate; Electromyographic: duration of vocalis muscle action potential; Cineradiographic: closing period of glottis | On average, the soft vocal attack has a longer rise time than the hard vocal attack. The pathological voices produce rise times similar to those of breathy voices. A soft vocal attack is also characterised by small initial air usage, inconspicuous preparatory muscle activity and a short adjusting period after glottal closure; hard by considerable air consumption, pronounced preparatory muscle activity and long adjusting period; breathy by air leakage prior to vocalization |
| Koster, Marx, Gemmar, Hess & Kunzel (1999): Qualitative and quantitative analysis of voice onset by means of a multidimensional voice analysis system (MVAS) using high-speed imaging | Acoustic, physiological, visual imaging | Not provided | Cross-sectional | 1 | Open quotient across two voice onset types (physiologic and breathy), glottal area analysis, EGG signal analysis, visual analysis of laryngoscopic images | Large inter and intra-subject variability across the dimensions of pre-phonatory closure, time span until achievement of full amplitude vibration and different open quotients |
| Kunduk (2004): Use of high-speed imaging to describe the voice initiation period in younger and older females | Acoustic, visual imaging | Definition provided for Voice Initiation Period (VIP), not voice onset in general (see Table 2 for VIP definition) | Cross-sectional | 40 | VIP | VIP is a valuable measure of vocal fold vibration in diagnosing age effects. Most timing characteristics of the vocal folds during the VIP were longer in the older group with some variables reaching the significance level. The presence of higher intra and inter subject variability in the older group suggests diminished efficiency in initiation and control of vocal fold vibration during the VIP |
| Kunduk, Yan, McWhorter & Bless (2006): Investigation of voice initiation and voice offset characteristics with high-speed digital imaging | Visual imaging | Definition provided for Voice Initiation Period (VIP), not voice onset in general (see Table 2 for VIP definition) | Cross-sectional | 2 | VIP and Voice Offset Period (VOP) | The younger subject achieved full glottal opening at a faster rate with rapid glottal area waveform (GAW) increase and less variation after the first four cycles compared to the older subject |
| Kunduk, Ikuma, Blouin & McWhorter (2017): Effects of Volume, Pitch, and Phonation Type on Oscillation Initiation and Termination Phases Investigated With High-speed Videoendoscopy | Visual imaging | Voice onset is defined according to the analysis approach taken in the study. Voice onset defined by stages:  Event I= vibration onset or first occurrence of VF vibration, detected when the glottal area waveform exhibited its first non-zero value  Event II= Vibration reaching full length  Event III= Vibration reaching full amplitude  Event IV= Loss of full-amplitude vibration.  Event V= Loss of full-length vibration.  Event VI= Vibration offset or last sign of VF vibration | Cross-sectional | 1 | Transient duration of the vibrating glottal length (T_len_), transient duration of the glottal area waveform (T_area_), Time offset between the beginning or end of the full-length vibration and full-amplitude vibration (T_Δ_), Variation of F0 during vocal fold oscillation initiation and termination segments (%PI) | Voice type effect was present for all variables during the initiation phase except for %PI (termination segment). During initiation, high-pitched phonation had shorter length and area transient durations than normal pitch when measured in the number of oscillation cycles. Loud phonation resulted in longer area transient duration than normal loudness phonation. The duration of T_Δ_ was shortest for the pressed phonation and the longest for high-pitched, loud phonation during the initiation phase. Pitch, loudness, and phonation types had significant effects on voice onset measures |
| Lebacq & DeJonckere (2019): The dynamics of vocal onset | Acoustic, aerodynamic, physiological, visual imaging | The process occurring between the first detectable glottal movement and the steady state vibration of the vocal folds; a dynamic phenomenon with a progressive adjustment of acting forces (lung pressure, intraglottal pressure, myoelastic tension of the VF oscillator generating the glottal impedance and inertance of the supraglottal vocal tract) until a steady state is reached | Cross-sectional | 1 | Characteristics of soft, breathy, and hard onset, Time course of oscillation amplitude, Number of cycles before the steady state, Pressure-glottal area relationship, Evolution of frequency during the first cycles | Soft onset is the most frequently observed onset type. Shape of glottis at onset, direction of vocal fold movement, amplitude of glottal area oscillations and closed phase described for each onset type. There are differences between soft and hard onsets for time course of oscillation amplitude. The onset phase has more cycles in the soft/breathy condition than in the hard condition. From the first onset cycles onwards, the intraglottal pressure during the opening phase exceeds that of the closing phase. There is a slight progressive decrease in F0 of vocal fold oscillation over the first cycles of breathy onset before a closed plateau is reached |
| Madill, Nguyen, McCabe, Ballard & Gregory (2019): Comparison of voice onset measures with glottal pulse identification in acoustic signals: preliminary analyses | Acoustic, airflow, physiological | The point where the vocal folds are adducted in readiness for subsequent phonation; the characteristics of which prime control of the vocal signal. Definitions for VAT, Vocal Rise Time (VRT), Voice Onset Coordination (VOC) and the first peak of the acoustic derivative (ADW1) also provided (see Table 2 for definitions) | Cross-sectional | 30 | Correlation between the time between the first acoustic deviation and ADW1 and existing voice onset measures of VAT, VRT and VOC | Correlation between ADW1 and voice onset measures are not consistent across measures and vowel types. Low correlation with VRT observed for /a/ only. Moderate correlation between ADW1 of /ou/ and VAT of /i/. ADW1 of /a/ had high correlation with VOC of /a/ and low correlation with VOC of /ou/. Moderate correlation between ADW1 and VOC for /ou/ |
| Maryn & Poncelet (2021): How Reliable Is the Auditory-Perceptual Evaluation of Phonation Onset Hardness? | Auditory-perceptual | Defined and classified in the three categories of soft, breathy and hard onsets | Cross-sectional | 20 | Reliability (intra and inter-rater) of auditory perceptual rating of phonation onset hardness | Variable within-rater reliability in evaluating voice onset hardness. Variable inter-rater reliability values, however this was low overall. Low proportional association between choices, significant differences between raters' choices of which two phonation onsets were harder |
| Mergell, Herzel, Wittenberg, Tigges & Eysholdt (1998): Phonation Onset: Vocal Fold Modelling and high speed glottography | Visual imaging | The amplitude growth of the vocal fold oscillation, which considers the voice as a product of the complete source-articulator system and phonation as the primary glottal signal | Solution validation with applied modelling and case example | 1 | Phonation Onset Time (POT) | Phonation onset could be described as a supercritical hopf bifurcation. Analyzing the two-mass model, the POT increases with decreasing subglottal pressure, increasing F0 and glottal rest area. A method is presented for the determination of the subglottal threshold pressure from POT measurements |
| Naghibolhosseini, Zacharias, Zenas, Levesque & Deliyski (2023): Laryngeal Imaging Study of Glottal Attack/Offset Time in  Adductor Spasmodic Dysphonia during Connected Speech | Visual imaging | Definition provided for Glottal Attack Time (GAT), not voice onset in general (see Table 2 for GAT definition) | Cross-sectional | 10 | GAT, Glottal offset time (GOT) | GAT was greater in the AdSD group than in  the normophonic group. More variability was observed in both GATs and GOTs amongst the AdSD group. The GAT and GOT time series were found to be nonstationary for the AdSD group while they were stationary for the normophonic voices. GAT can help function as measure to discriminate normophonic speakers from those with AdSD in connected speech contexts |
| Orlikoff, Deliyski, Baken & Watson (2009): Validation of a Glottographic Measure of Vocal Attack | Acoustic, physiological, visual imaging | The speed with which the vocal folds adduct to the midline including two distinct phases; 1) the pre-phonatory adjustment phase, associated with setting the appropriate tension, gross adduction and aerodynamic forces, and 2) the attack phase associated with the onset of vocal fold oscillation and sound generation | Cross-sectional | 5 | VAT | In breathy attack, VAT ranged from 7.6ms- 38ms. VAT values for comfortable onsets were shorter, ranging from -1.4ms to 9.6ms. In hard glottal attack, VAT was negative, ranging from -9.5ms to -1.7ms. There was strong and direct correlation between VAT and digital kymographic data |
| Patel (2016): Vibratory onset and offset times in children: A laryngeal imaging study | Visual imaging | The time interval between the first instance of vibratory motion to the beginning of steady-state phonation | Cross-sectional | 86 | Vibratory onset time, Vibratory offset time. | Children exhibited significantly shorter vibratory onset and offset times compared to adult females and adult males). No significant difference between adult females and males for vibratory onset time. No significant main effect for waveform type and no significant interaction between group and waveform type. |
| Patel, Walker & Dollinger (2017): Oscillatory Onset and Offset in Young Vocally Healthy Adults Across Various Measurement Methods | Visual imaging | Comprises of various transitory segments, being 1) vocal fold adductory phase of approximately 203± 147ms, followed by 2) small irregular vocal fold edge oscillatory motion for a brief duration of time (80± 43ms) until steady-state cyclic oscillations are achieved | Cross-sectional | 71 | Oscillatory Onset Time (OOT), VIP, POT, Oscillatory Offset Time (OOT_off_) and Voice Offset Period (VOP_off_) | Normative values are presented for OOT and OOT_off_ for normophonic male and female voices producing /hi/ syllable trains. A positive relationship was observed between VIP and POT and OOT_off_ and VOP_off_. A non-linear relationship was found between OOT, VIP and POT |
| Patel, Forrest & Hedges (2017): Relationship Between Acoustic Voice Onset and Offset and Selected Instances of Oscillatory Onset and Offset in Young Healthy Men and Women | Acoustic, visual imaging | Not provided (however some definitions of measures not explored in this study are defined) | Cross-sectional | 56 | Onset of first oscillatory motion (X1g), First medial contact of the vocal folds (X1.5g), Onset of sustained oscillations (X2g), First instance of oscillatory offset (X3g), First incomplete vocal fold closure (X3.5g), Complete cessation of oscillatory motion (X4g), Onset of the acoustic signal (X1a), Offset of the acoustic signal (X2a) | The onset of the acoustic signal is closely related to the first medial contact of the vocal folds, but there is a longer latency for this in women as compared to men. The offset of the acoustic signal occurs immediately after glottal adduction |
| Peters, Boves & van Dielen (1986): Perceptual judgment of abruptness of voice onset in vowels as a function of the amplitude envelope | Acoustic, auditory perceptual | Not provided | Cross-sectional | 4 | Product-moment correlations between acoustic measures of voice onset; averaged perceptual ratings of abruptness of voice onset | Moderately high inter-rater reliability between speech pathologists. The abruptness of voice onset is best predicted by the rise time of the amplitude envelope |
| Plant, Freed & Plant (2004): Direct measurement of onset and offset phonation threshold pressure in normal subjects | Acoustic, airflow, physiological | Definition provided for Phonation Threshold Pressure (PTP), not voice onset in general (see Table 2 for PTP definition) | Cross-sectional | 5 | Relationships between the variables of F0, intensity, closure speed of the vocal folds and laryngeal airway resistance and phonation threshold pressure, Differences in measures between onset and offset PTP | The largest number of significant relationships occurred in relation to intensity and F0. Vocal intensity, airway resistance, F0 and the first derivative of the EGG signal significantly contributed to the prediction of phonation threshold pressure contributed. |
| Roark, Watson & Baken (2012): A Figure of Merit for Vocal Attack Time Measurement | Acoustic, physiological | Not provided | Validation study with cross-sectional data | 112 | Pearson correlation as a companion metric of VAT's fidelity | The median correlation coefficient was 0.975 for 1033 VAT measures, with only 2.8% of values rejected. Pearson´s correlation coefficient can be used to measure the quality of VAT measurement |
| Roark, Watson, Baken, Brown & Thomas (2012): Measures of Vocal Attack Time for Healthy Young Adults | Acoustic, physiological | Definition provided for VAT, not voice onset in general (see Table 2 for VAT definition) | Cross-sectional | 112 | VAT | Normative values of VAT from young people were reported with a mean VAT of 1.98ms |
| Shiba & Chhetri (2015): Dynamics of Laryngeal Posturing at Phonation Onset | Acoustic, visual imaging | The time from the onset of glottal adduction to production of the acoustic signal | Cross-sectional | 27 | POT, Final Phonatory posture time (FPT), Phonatory Posture Time (PPT), F0 - stabilization time, Posture Characteristics at Phonation Onset % occurrence across samples | Average FPT, PPT, and POT values are reported for different onset types. The following posturing features were observed: 1) pressed phonation: increased speed of closure just prior to final posture, complete glottal closure, and increased supraglottic hyperactivity; and 2) breathy phonation: decreased speed of closure prior to final posture, increased posterior glottal gap, and increased mid-membranous gap |
| Simon & Maryn (2022): Can the reliability of auditory-perceptual assessment of voice onset hardness by speech and language pathology students be improved thanks to training? | Auditory-perceptual | The phase occurring between the first oscillatory movement of the vocal folds and the moment they reach steady state vibration. A dynamic phenomenon with a progressive adjustment of acting forces such as subglottal pressure, intraglottal pressure, myoelastic tension of the vocal folds, supraglottic articulators, etc. It is categorised into three types of phonation onset hardness: soft, breathy and hard | Cohort | 11 | Reliability (intra and inter-rater) of auditory perceptual rating of phonation onset hardness | Inter- and intra-rater reliability did not improve following training, for neither nominal nor continuous voice onset type assessments (see Table 7 in manuscript) |
| Tigges, Wittenberg, Mergell & Eysholdt (1999): Imaging of vocal fold vibration by digital multi-plane kymography | Visual imaging | Not provided | Cross-sectional | 1 | Kymographical characteristics of phonation onset, aperiodic vibration and anterior-posterior mode of vibration | Different types of phonation onset are described according to what is visible through digital multi-plane kymography across the different stages of voice onset |
| Watson, Freeman & Dembowski (1991): Respiratory/ laryngeal coupling and complexity effects on acoustic laryngeal reaction time in normal speakers | Acoustic | Definition provided for LRT, not voice onset in general (see Table 2 for LRT definition) | Cross-sectional | 30 | Laryngeal Reaction Time (LRT) | LRT values were measured across tasks with differing vocal fold configurations associated with sound production (referred to as ‘respiratory/laryngeal coupling’). The range of LRT values from shortest to longest time was 69ms. Significant differences were identified across tasks, suggesting LRT is sensitive to both respiratory/laryngeal coupling and complexity of vocal task |
| Watson, Baken, Roark, Reid, Ribeiro & Tsai (2013): Effect of Fundamental Frequency at Voice Onset on Vocal Attack Time | Acoustic, physiological | The movements of the larynx leading to and including the initiation of tone, including the glottal shock, breathed and simultaneous vocal attacks | Cross-sectional | 13 | VAT | Findings reveal an association between VAT and increases in vocal fold tension associated with  the production of high rates of vocal fold vibration |
| Watson, Baken & Roark (2016): Effect of Voice Onset Type on Vocal Attack Time | Acoustic, physiological | The speed with which the vocal folds adduct to the midline | Cross-sectional | 112 | VAT of aspirated and unaspirated vocal productions | There is a significant effect of onset type on VAT, with the mean VAT for the ‘‘hallways’’ (aspirated) task greater than the mean VAT for the sustained /ɑ/ and ‘‘always’’ (unaspirated) tasks. Mean VAT for /ah/: 1.89 ms; Mean VAT for "Always" (unaspirated): 1.83 ms; Mean VAT for "Halllways" (aspirated): 5.98 ms |
| Werner-Kukuk & Von Leden (1970): Vocal Initiation: High Speed Cinematographic Studies on Normal Subjects | Visual imaging | A function of vocal cord placement at the start of phonation which in turn depends on the precise coordination between the subglottic pressure and the resistance at the level of the larynx | Cross-sectional | 4 | Measurements taken between ventricular folds, vibrating vocal folds at anterior, middle and posterior thirds, Open quotient, Speed quotient | Marked differences were confirmed across three onset types for vocal fold closure (soft: complete, hard: firmly closed prior to phonation, breathy: no tendency to approximate), amplitude (soft: increases gradually, hard: increases rapidly and breathy: irregular), open quotient (soft: 0.57, hard: 0.87, breathy: 0.82) and speed quotient (soft: 0.90, hard: 1.6, breathy: 0.83). Typical vibration patterns exist across different means of voice initiation |
| Wittenberg, Moser, Tigges & Eysholdt (1995): Recording, processing, and analysis of digital high-speed sequences in glottography | Visual imaging | Not provided (only present in result section following data acquisition, not prior to inform data collection) | Solution validation with cross-sectional data | 30 | Speed, acceleration rates, F0, amplitude and perturbation parameters | Vocal fold closure patterns, time of glottal closure and vibrational onset outlined for hard, normal and soft onset types. Values for soft, normal and hard onsets were provided for male and female participants for two vowel productions |
| Wittenberg, Mergell, Tigges & Eysholdt (1997): Quantitative characterization of functional voice disorders using motion analysis of high speed video and modeling | Visual imaging | Definition provided for POT, not voice onset in general (see Table 2 for POT definition) | Solution validation with cross-sectional data | 52 | POT, F0 of subjects | Mean pitch of female patients (240-320Hz) was higher than the mean pitch of the male group; mean pitches for both sexes higher than normal speech frequency (200Hz in females, 125Hz in males) and mean frequency of hyperfunctional males is higher than the mean pitch of hypofunctional males (205Hz vs 190Hz). The lower boundary for onset time of hyperfunctional females and hypofunctional males is provided. POT has error range of 50-100% while pitch error is ~5% |
| Wittenberg, Tigges, Mergell & Eysholdt (2000): Functional imaging of vocal fold vibration: Digital multislice high-speed kymography | Visual imaging | Not provided | Review paper with case example | 1 | Kymographical characteristics of phonation onset, aperiodic vibration and vibration mode, kymographical differences between laryngeal high-speed imaging and digital strobolaryngoscopy | Differences in pre-phonatory adduction movements, glottal closure patterns, the onset of vocal fold vibration and achievement of steady state phonation outlined across normal, hard and breathy onset types |
