# Supplementary material for: Vowel onset measures and their reliability, sensitivity and specificity: A systematic literature review
Source: PLoS One. 2024 May 2;19(5):e0301786. doi: 10.1371/journal.pone.0301786 (PMC11065290; doi:10.1371/journal.pone.0301786)
Supplement: Attachment — Submitted filename: Response to Reviewers.pdf [file pone.0301786.s005.pdf]

February 25, 2024

Response to Reviewer Comments

Re: Submission number: PONE-D-23-27828
Title: Vowel onset measures and their reliability, sensitivity and specificity: A systematic literature review

Dear Dr Maniaci,

Thank you kindly for advising us of the additional journal requirements when submitting our revision, and for sharing the points raised during our paper's review process. We have tabulated our responses to both the journal requirements stipulated and the comments provided by the reviewers below:

Comment provider	Comment	Response
Editor/ Journal requirements	Please ensure that your manuscript meets PLOS ONE's style requirements, including those for file naming. The PLOS ONE style templates can be found at: https://journals.plos.org/plosone/s/file?id=wjVg/PLOSOne_formatting_sample_main_body.pdf and https://journals.plos.org/plosone/s/file?id=ba62/PLOSOne_formatting_sample_title_authors_affiliations.pdf	Thank you for bringing our attention to these resources. Our manuscript and associated files have been updated to reflect the style requirements specified in these documents.
	Did you know that depositing data in a repository is associated with up to a 25% citation advantage (https://doi.org/10.1371/journal.pone.0230416)? If you've not already done so, consider depositing your raw data in a repository to ensure your work is read, appreciated and cited by the largest possible audience. You'll also earn an Accessible Data icon on your published paper if you deposit your data in any participating repository (https://plos.org/open-science/open-data/#accessible-data).	Thank you for providing this recommendation. We have added our core data (being the summary of literature extracted from all 35 studies involved in our review) to the OSF Home repository (line 188). The DOI and citations are as follows: DOI 10.17605/OSF.IO/N65SX; Chacon, A. M. (2024, February 17). Vowel Onset Reliability, Sensitivity and Specificity. Retrieved from osf.io/n65sx

	Thank you for stating the following in the Acknowledgments Section of your manuscript:<br>*"Contributions by Michael Döllinger were supported by Deutsche Forschungsgemeinschaft (DFG) under grant no. DO1247/8-2. Contributions by Tomás Arias-Vergara were supported by Deutsche Forschungsgemeinschaft (DFG) under grant no. SCHU3441/3-2. Contributions by Antonia Margarita Chacon and Duy Duong Nguyen were supported by the Doctor Liang Voice Program at The University of Sydney."*<br><br>Funding information should not appear in the Acknowledgments section or other areas of your manuscript. We will only publish funding information present in the Funding Statement section of the online submission form. Please remove any funding-related text from the manuscript and let us know how you would like to update your Funding Statement. Currently, your Funding Statement reads as follows:<br>*"Contributions by MD were supported by Deutsche Forschungsgemeinschaft (DFG) under grant no. DO1247/8-2. Contributions by TA were supported by Deutsche Forschungsgemeinschaft (DFG) under grant no. SCHU3441/3-2. Contributions by AC and DN were supported by the Doctor Liang Voice Program at The University of Sydney. The funders had no role in study design, data collection and analysis, decision to publish, nor preparation of the manuscript."*<br><br>Please include your amended statements within your cover letter; we will change the online submission form on your behalf.	Thank you for your explanation and for bringing this to our attention. The funding information has now been removed from the Acknowledgements section. Our finalised funding statement is provided in our cover letter and below:<br><br>*'Contributions by MD were supported by Deutsche Forschungsgemeinschaft (DFG) under grant no. DO1247/8-2. Contributions by TA were supported by Deutsche Forschungsgemeinschaft (DFG) under grant no. SCHU3441/3-2. Contributions by AC and DN were supported by the Doctor Liang Voice Program at The University of Sydney. The funders had no role in study design, data collection and analysis, decision to publish, nor preparation of the manuscript.'*
	We note that you have referenced (ie. unpublished dissertations/works) which has currently not yet been accepted for publication. Please remove this from your References and amend this to state in the body of your manuscript: (ie "Bewick et al. [Unpublished]") as detailed online in our guide for authors:	Thank you for advising us of this. We reviewed our reference list and identified the theses by Kunduk and Simon & Maryn and the conference presentation by Madill and colleagues to be the only unpublished works

	http://journals.plos.org/plosone/s/submission-guidelines#loc-reference-style	referred to throughout our manuscript. We have amended the references to these papers as [Unpublished] throughout our manuscript as advised.<br><br>However, please note that these three unpublished works formed part of the 35 studies included in our review. As such, we still need to refer to these throughout our manuscript, however, were uncertain as to how best to stylistically present these. While the format of "Bewick et al. [Unpublished]" presented well in our tables, we were not sure how to record this when referring to the unpublished work in the manuscript body, both in isolation and when presented with published articles (i.e., use of rounded vs square brackets, etc.). We checked the PLOS One submission guidelines but could not find any recommendations for this (References - Submission Guidelines), so we contacted the PLOS One office (Case Number: 08400370) but did not receive clear advice regarding how this should be presented. As such, we've attempted to present this in a manner which seemed clear, by retaining the square bracketing format throughout the manuscript, but mentioning {Unpublished} in braces within these. Please advise if this needs to be presented according to a different format and we will happily amend this throughout.

	We note you have included a table to which you do not refer in the text of your manuscript. Please ensure that you refer to Tables 3, 5 and 6 in your text; if accepted, production will need this reference to link the reader to the Table.	Thank you kindly for bringing this to our attention. These tables have now been referred to in the appropriate sections of our manuscript.
	We note that this manuscript is a systematic review or meta-analysis; Please upload copies of the completed PRISMA checklist as 'Supporting Information' with a file name "PRISMA checklist".	Thank you for bringing this to our attention. We had attached the PRISMA checklist in our prior submission without considering that it should be provided as a supporting information file. This has now been referred to in the manuscript text as a supplementary material and re-uploaded as a supporting information file.
	Please review your reference list to ensure that it is complete and correct. If you have cited papers that have been retracted, please include the rationale for doing so in the manuscript text, or remove these references and replace them with relevant current references. Any changes to the reference list should be mentioned in the rebuttal letter that accompanies your revised manuscript. If you need to cite a retracted article, indicate the article's retracted status in the References list and also include a citation and full reference for the retraction notice.	We have re-done the referencing for the entire manuscript and updated the reference list using the Endnote platform. We've also checked this to ensure it is complete and correct. The only references which have been revoked from the reference list are those referring to unpublished works, as per your above advice.
Reviewer 1	Well written manuscript. Well done to all authors. I would just like to highlight minor comments.<br><br><u>Methods</u><br>Line 118- I would suggest to put MEDLINE via OVID, EMBASE via OVID	We thank Reviewer 1 for their congratulatory comments.<br><br><u>Methods commentary</u><br>Thank you for this suggestion. This change is now reflected in the manuscript text (line 134).
	<u>Study population characteristics</u><br>Line 204- I would suggest putting these summarized details in a table 1, it would be easier to read. Don't have to repeat them in text.<br>Line 518- above mentioned.	<u>Study population commentary</u><br>Line 204: Thank you for your suggestion. Our team agrees that a summary table allows for this information to be presented more clearly. As we already had a Table 1 summarising

		study characteristics for each individual study, we have added a Table 2 to present these summarised details and deleted the original text.<br><br>Line 518: We thank the reviewer for noting this. This section relates to ‘Research Quality’, encompassing the results of reliability, sensitivity and specificity analysis. Given that this section contains several paragraphs detailing how reliability, sensitivity and specificity were explored across the dataset, we thought a summative sentence was warranted to consolidate our findings for the reader. Furthermore, as our research question relates to examining reliability, sensitivity and specificity, we thought that a summative sentence would serve to improve the reader’s comprehension of our findings. Our research team finds this sentence to be of value in functioning as a brief summative statement, however, please advise if you think it significantly impacts the readability of this section and should be excluded.
	Supplement materials<br>Please include search strategy for all the databases.	Supplement materials commentary<br>Thank you for bringing to our attention that the search strategy provided did not completely reflect the strategy adopted for two search engines used (MEDLINE and EMBASE). We have adapted our supplementary materials accordingly. The

		same search strategy was otherwise used for all remaining search engines. Given that both search strategies were replicated across several databases, only two have been provided to cover the breadth of strategies applied in our search process.
Reviewer 2	Abstract<br>- Purpose: To systematically review validity of vowel onset measures for clinical voice assessment.<br>- Methods: Electronic databases searched for studies measuring vowel onset using >1 instrument. Data extracted on measures, reliability, sensitivity, specificity.<br>- Results: 35 studies covered 5 measurement categories; 39 measures identified. Measures showed low-to-moderate reliability; sensitivity rarely assessed.<br>- Conclusions: Heterogeneity in populations and methods preclude conclusions on most valid measures. Standardization of research methodology is needed	We thank Reviewer 2 for their comprehensive comments, which we believe has helped develop this work into a manuscript of higher quality.<br><br>Abstract commentary<br>Thank you for these contributions. We believe this adds to the clarity of the abstract summary and we value your suggestions. These recommendations have been integrated into our abstract (lines 27-50).
	Introduction<br>1. Note prevalence of voice disorders and importance of early/accurate diagnosis<br>2. Outline current clinical assessment methods and limitations (perceptual only)<br>3. Define voice onset and importance in assessment/treatment monitoring<br>4. Highlight variability in instrumental onset measures used across studies<br>5. State importance of establishing valid clinical measures for standardized assessment<br>6. Explicitly state research questions:<br>a) What vowel onset measures have been used instrumentally?<br>b) What is the evidence for their reliability, sensitivity and specificity?<br>7. Hypothesize most effective measures will demonstrate high reliability, sensitivity, specificity	Introduction commentary<br>1. This has been added to lines 53-55 of the Introduction section.<br><br>2. Current clinical assessment methods are now outlined in lines 57-60, with the limitations of these pertaining to voice onset highlighted in the pre-existing text of lines 61-62.<br><br>3. Voice onset is defined in lines 66-71. Its importance in assessment/treatment monitoring is provided in lines 62-65.

	8. Aim to inform standardized assessment by identifying valid instrumental onset measures	4. Thank you for this suggestion. This is mentioned in lines 98-111.<br><br>5. Thank you for your suggestion. This information has now been added in lines 116-119.<br><br>6. Thank you for considering our research question and dividing it into two, clear research questions. However, according to the Cochrane Handbook 1.1, a systematic review should only be based on a single, specific research question. Likewise, the use of our single question informed our search strategy, and outlining our question as being two-fold may have involved our team taking a slightly different approach to our search strategy. As such, to remain consistent with the guidelines and to ensure our manuscript reflects the exact research approach taken for replicability purposes, our team agrees that it is best to retain our single research question as originally stated.<br><br>7. Thank you for this suggestion. A hypothesis statement has now been integrated into lines 120-122.<br><br>8. This recommendation has been integrated into lines 116-119.
	<u>Methods</u><br>1. Specify retrospective systematic review design	<u>Methods commentary</u><br>1. Thank you for this suggestion. The design

	2. Provide inclusion/exclusion criteria in detail<br>3. Describe all information sources and search strategy used<br>4. Explain screening, data extraction and analysis processes<br>5. Detail all variables/data extracted from studies<br>6. Describe approach to risk of bias/quality assessment<br>7. State programs/statistical analyses used	has now been specified in line 128 of the manuscript.<br><br>2. All inclusion/ exclusion criteria applied to our search strategy is outlined in the section titled 'Inclusion Criteria', lines 151-158. Please advise if you feel that any specific further detail is required here.<br><br>3. All information sources are outlined in lines 134-136. The search strategies applied across these databases is referred to in lines 138-149 and is further detailed in S3.<br><br>4. The screening process is outlined in lines 163-167. Further information pertaining to data extraction has been added in lines 185-188. Further information pertaining to analysis is provided in lines 190- 194.<br><br>5. Thank you for this suggestion. We extracted many variables and believed that listing these would not be the most effective means of presenting this information. As such, the variables used to inform data extraction are provided in our data extraction table in S4.<br><br>6. This is summarised in lines 196-209, with some additional information added in lines 199-201 to further clarify the assessment approach.

		7. The only programs used for the purposes of our systematic review were Covidence (referenced in line 165) and the GRADEPro application (referenced in line 209) to facilitate the risk of bias/ quality assessment rating. As stated in lines 188-189, due to heterogeneity in the data reported across studies, no statistical analysis was conducted and as such, no statistical analysis programs were used.
	<u>Results</u><br>1. Report study selection details quantitatively in PRISMA flow diagram<br>2. Provide characteristics of included studies as evidence tables<br>3. Categorize measures used and define each clearly<br>4. Present findings for each measurement category separately:<br>a) Report measures used, populations, reliability, etc. numerically<br>b) Include relevant tables/figures to organize data<br>c) Note any similarities/differences between categories<br>d) Acknowledge limitations like heterogeneity between studies	<u>Results commentary</u><br>1. Quantitative details have already been integrated into our PRISMA flow diagram as per the PRISMA statement. These details are provided, in brackets, following each instance in our study methodology where papers were excluded. As our present figure aligns with the format specified by the PRISMA guidelines, we want to ensure our diagram acts in accordance with these standards and does not contain any extraneous information. If we have misinterpreted your comment here, please advise what other information is required as per the PRISMA guidelines and we'll be sure to include this.<br><br>2. Thank you for this suggestion. We certainly acknowledge the value of evidence tables for treatment-based systematic reviews. However, much of the novel information which this table would introduce is not

		relevant for the purposes of our research question. Evidence tables tend to prove most useful when reviewing papers pertaining to patient treatment outcomes; hence the inclusion of the columns of 'Magnitude of Benefit', 'Absolute Risk Reduction' and 'Number needed to treat'. Our paper is focused on assessment methods for vowel onset, and hence many of these columns become redundant for our purposes. The evidence table columns of relevance to our review (i.e., study design, quality of study, etc.) are already summarised in our existing Table 1.<br><br>3. Each measure used has been defined in Table 3 (Summary and definition of voice onset measures). Each measure is categorised into one of five voice onset measurement types, being auditory perceptual, acoustic, aerodynamic, physiological and/or visual imaging. If you think we are missing pertinent information pertaining to the definition of particular vowel onset measures or their categorisation, please provide additional information regarding this and we'll feel better equipped to act upon your suggestion.<br><br>4. Thank you for this suggestion.<br>A) Please note that 'measures used' are already provided in each measurement

		category table. The populations pertaining to each paper is summarised in Table 1. It is our understanding that papers should seek to avoid any replication of information, so our team agrees that retaining population details in Table 1 rather than repeating this for each measurement category is an appropriate approach. For ease of readability, we believe that continuing to present the reliability, sensitivity and specificity information- particularly as this relates to a core element of our research question- together in a single table is the clearest option to present this data for our readers.<br><br>B) Our team has done our best to organise the range of data collected in the existing tables present in our manuscript results section. If you believe there is clearly information lacking or a need for particular figures to be developed, we would value further direction as to which specific tables and figures would help to further organise and present our data.<br><br>C) Thank you for your suggestion.<br>The major similarities and differences across the papers which are pertinent to our research question are summarised in the sections from lines 473 to 574. However, we did not consider this in light of our measurement categories. This information has now been integrated throughout these sections (i.e.

		automation, reliability, sensitivity and specificity and GRADE analysis).<br><br>D) This discussion pertaining to limitations throughout our research process is provided within the discussion section. Are you suggesting that we introduce a new section towards the end of our results section which discusses the factors which limited what conclusions we were able to draw and report in our results? If so, please advise us if you think this is necessary. Otherwise, to save us from rehashing the discussion of limitations including heterogeneity (which has its own dedicated section in our discussion), our team thinks it may be best to retain the formatting of this as it currently presents.
	<u>Discussion</u><br>1. Compare findings to other reviews on this topic and highlight novel insights<br>2. Critically discuss sources of heterogeneity between studies (measures, populations, etc.)<br>3. Analyze how heterogeneity limits interpretation and generalizability<br>4. Explore possible reasons for low/variable reliability reported<br>5. Address lack of sensitivity/specificity analyses between studies<br>6. Relate findings to current clinical practices and limitations<br>7. Recommend standardized measurement criteria/protocols for future research<br>8. Suggest priorities for future studies based on gaps identified<br>E.g. Validating individual measures, diverse populations/disorders,	<u>Discussion commentary</u><br>1. Thank you for this suggestion; we agree that comparing our findings to that of similar studies would be an ideal means of highlighting our novel insights. However, as outlined in lines 729-732, we have been unsuccessful in sourcing any reviews of a similar nature. We have just conducted an updated search and still find that no other reviews on the topic of vowel onset measures and their reliability, sensitivity and specificity is available to enable our comparison.<br><br>2. Thank you for suggesting this valuable

	automation<br>9. Discuss implications of findings for voice assessment standardization<br>10. Conclude by emphasizing need for high-quality data before recommending measures<br>11. Acknowledge limitations like excluding non-English sources<br>12. Consider strengths like comprehensive search and inclusion of gray literature	addition to our discussion. This information has now been added in lines 630-642.<br><br>3. We have now included an analysis of how heterogeneity limits interpretation and generalisability in lines 643- 651.<br><br>4. This exploration has now been added in lines 692- 703. Thank you for suggesting the addition of this information to our reliability discussion.<br><br>5. The lack of sensitivity and specificity analyses has now been addressed in lines 706-709.<br><br>6. Thank you for this recommendation; we agree this is an important element to add to our discussion. A section relating to this has now been added in lines 780- 791.<br><br>7. This suggestion has been added to lines 761-763.<br><br>8. Our team has sought to integrate your suggestions into our section of future research priorities from lines 757- 777. Thank you for recommending these.

		9. We have now integrated this valuable point into lines 751-754, as part of our newly added section on ‘clinical implications’.<br><br>10. Thank you for this recommendation. This has been added into our conclusion section, in line 784.<br><br>11. This was already stipulated in lines 712-726, however we’ve added some further detail here to make this clearer.<br><br>12. Thank you for this suggestion. This has been further highlighted in our ‘strengths and limitations’ section, in lines 713-714.

We greatly appreciate the time taken to review our manuscript. We look forward to hearing from you in due course.

Kind regards,

Antonia Chacon, Duy Duong Nguyen, John Holik, Michael Döllinger, Tomás Arias-Vergara and Catherine Madill
